# Amaranth as a natural food colorant source: Survey of germplasm and optimization of extraction methods for betalain pigments

**DOI:** 10.3389/fpls.2022.932440

**Published:** 2022-09-21

**Authors:** Jay E. Howard, Maria B. Villamil, Chance W. Riggins

**Affiliations:** Department of Crop Sciences, University of Illinois at Urbana-Champaign, Urbana, IL, United States

**Keywords:** betalains, plant pigments, natural colorants, *Amaranthus*, germplasm diversity

## Abstract

Growing consumer demands for healthier foods have evoked trends in the food industry to replace synthetically produced colorants with naturally derived alternatives. Anthocyanins currently comprise the bulk of the natural colorant market, but betalains offer advantages where anthocyanins have limits. *Amaranthus* species are appealing betalain sources given their extensive pigmentation patterns and recognized food status around the world. An advantage of amaranths as natural food colorants is that, when grown as leafy vegetables, water extracts would be compliant with U.S. Food and Drug Administration guidelines as “vegetable juice” colorants. Thus, we developed a methodology based on U.S. FDA guidelines to investigate betalain diversity among forty-eight amaranth accessions grown as leafy vegetables. Total betacyanin concentrations ranged from 4.7 to 478.8 mg/100 g dry weight, with amaranthin and isoamaranthin identified as major constituents. Our findings will guide future research on amaranths to determine economic viability and suitability for growing natural colorant markets.

## Introduction

The use of naturally derived food and beverage colorants has grown substantially in recent years. By 2024, the global natural colors market is estimated to surpass 2 billion USD (Ahuja and Rawat, 2018). Despite the stability, consistency, and low cost of synthetic food dyes, many companies have begun searching for natural replacements due to consumers’ wariness of their unnatural origin and potential adverse health effects. Synthetic azo dyes are widely used in food industry because of their stability, vivid colors, and relative low cost of production compared to natural colorants. FD&C Red 40 (Allura Red AC) is one of the most popular synthetic azo colorants. More than 12 million pounds were certified for use in the United States alone in 2019–2020 (FDA, 2021). Previous studies, however, have associated the consumption of certain synthetic azo dyes with hyperactivity in children leading to required precautionary label statements in the EU and an accelerated shift to natural alternatives in the food industry around the world (Amchova et al., 2015; Oplatowska-Stachowiak and Elliott, 2017).

Anthocyanins and betalains are the most commonly used natural alternatives to synthetic reds, with anthocyanins typically performing best in high acidity applications (pH < 4) and betalains performing best at pH levels above 4. Anthocyanins are commercially sourced from many crops such as grapes, carrots, and corn, while betalains are exclusively sourced from red beets (Reinhold and Schweiggert, 2016). Other crops such as pitaya, prickly pear, and amaranth have been suggested as potential sources of betalains, yet lack of research and uncertain agronomic potentials have limited their commercial application (Gengatharan et al., 2015). As seen with other natural colorants, utilizing alternative crops for betalain pigments would expand the geographic ranges of production and potentially introduce color extracts with novel hues, improved stability, and enriched sensory characteristics such as taste and odor.

Interest in amaranths as resilient and healthy alternative crops continues to gain traction (Zhang et al., 2018). Amaranths possess favorable agronomic characteristics (e.g., rapid growth rates, C4 photosynthesis, and high tolerance to drought, heat, and salinity) making them appealing crops for agriculturally marginal lands (Sarker and Oba, 2018a,b, 2020). Cultivated amaranths are also notable for their high nutritional value, palatability, and versatility as ingredients for an expanding market of food and cosmetic products (Velarde-Salcedo et al., 2019). Both amaranth seeds and leaves have diverse nutritional profiles—most notably high in protein, vitamin C, lysine, iron, and fiber (Rastogi and Shukla, 2013). Unlike many cereals, amaranth seeds are gluten-free (Schoenlechner et al., 2010). The seeds can be boiled, roasted, or popped to create a standalone meal or add a unique flavor to other foods. Most amaranth species are considered edible, but three species are commercially cultivated worldwide as pseudocereal crops, namely *A. caudatus*, *A. cruentus*, and *A. hypochondriacus*. Two additional amaranths, *A. tricolor* and *A. blitum*, are also grown as leafy vegetables mainly in Africa and Asia.

One historical use of amaranth not often discussed is its use as a source of natural colorants. Aztec, Incan, Hopi, and other civilizations indigenous to the Americas used colored amaranth leaves or inflorescence tissues as a source of red dye for food, textiles, and religious ceremonies (Sauer, 1967). The fascination with the purple-red pigments produced by amaranth continues today and has led to the continued selection of many different cultivars displaying intense coloration and/or intricate patterning from around the world. Amaranth pigments are betalains, a class of pigments restricted in distribution among plants to the Caryophyllales order (Sarker et al., 2022), several fungi genera, and most recently, the bacterium *Gluconacetobacter diazotrophicus* (Contreras-Llano et al., 2019). Betalains are subclassified based on hue into magenta-appearing betacyanins and betaxanthins, which appear yellow-orange. Amaranth betacyanins are composed mostly of amaranthine and isoamaranthine and of lesser amounts of betanin and isobetanin (Sarker and Oba, 2021). Unlike the more prominent anthocyanin-based pigments derived biosynthetically from phenylalanine, betalains are nitrogenous compounds derived from tyrosine. Betalains typically show much greater pH stability than anthocyanins which has led to their widespread use in the food industry as a natural colorant for products with a pH value greater than 3 (Reinhold and Schweiggert, 2016).

Formal studies of amaranth as an alternative to beet-derived colorants began in the 1980s (Huang and Von Elbe, 1986). Amaranth’s low maintenance requirements, rapid growth rate, and high biomass yields obtained in various environments make it an attractive alternative to beets (Teutonico and Knorr, 1985). In addition, amaranth extracts contain less earthy off-odors typical of beet extracts. The primary betacyanin compound in *Amaranthus* extracts is amaranthin (betanidin 5-O-β-glucuronosyl-glucoside), whereas betanin (betanidin 5-O-β-glucoside) is the major betacyanin in beets (Huang and Von Elbe, 1986). Betalains from amaranth could potentially be extracted from leaf and stem tissue at a juvenile growth stage or from the remaining forage after seed harvest. The former option seems to be the best suited for color extraction; when harvested as an edible vegetable, the resulting extracts would face fewer regulatory hurdles. In the United States, water extracts of amaranth greens would fit under the guidelines for “vegetable juice” colorants exempt from certification and could be simply labeled as “vegetable juice for color” (Sec. 73.260 of the Code of Federal Regulations, Title 21). Betacyanin pigments from *Amaranthus* species are already approved as a natural food colorant in China (Hygienic Standards for Food Additives, GD2760-2014).

The broadest surveys of *Amaranthus* germplasm to determine the diversity of betacyanin content and composition were conducted two decades ago (Cai et al., 1998a, 2001a). These studies included cultivated and wild species and, though providing useful baseline information on betalains, were limited by ambiguous growing conditions, incongruent selection of tissue types, and extraction methods that are not high throughput. In this study, we reinvestigate previously examined genotypes and uninvestigated new varieties of amaranths specifically bred for intense pigmentation sourced from the U.S. National Plant Germplasm System and commercial suppliers with the objective to identify lines with the highest pigment yields from vegetative tissue. Unlike previous studies, our methodology was designed to optimize precise, high throughput extraction protocols of uniformly grown and harvested amaranth vegetative material that aligns with strict U.S. FDA food additive guidelines and current industry standards. Findings from this survey provide new information on betalains among diverse amaranth species and will support future research of amaranths as potential sources of natural colorants with novel color hues compared to other betalain-producing alternatives.

## Materials and methods

### Genotype selection and growing conditions

Our selection of plants was guided in part by FDA natural colorant guidelines (CFR Title 21 73.260) and practical considerations for identifying top pigment producers in greenhouse screens as a first step for eventual field trials. Although some amaranths have densely pigmented inflorescences, this part of the plant typically is not consumed (seeds excluded) and therefore would require FDA approval as a colorant source. Alternatively, when grown as a leafy vegetable, amaranth could be harvested multiple times throughout the growing season and extracts would be FDA compliant as a “vegetable juice colorant.” For this study, we use the term ‘vegetable amaranth’ to represent all amaranth species we analyzed at the same vegetative growth stage. We selected forty-eight *Amaranthus* accessions for examination acquired from the USDA North Central Regional Plant Introduction Center in Ames, Iowa and commercial suppliers (Table 1). The majority of cultivated amaranth germplasm curated by the USDA National Plant Germplasm System sources back to collection and breeding initiatives by the Rodale Institute starting in 1976 (Brenner et al., 2000). Our selection also included several non-pigmented biotypes of commonly grown cultivated amaranths for comparison, in addition to a few wild or weedy amaranth species (e.g., *A. quitensis*, *A. graecizans*, and *A. dubius*) for which little or nothing of betalains are known.

**Table 1:** Summary of betacyanin content, composition, and spectral qualities of all 48 accessions and genotypes surveyed. Accessions are ranked in order of highest total betacyanin yield in mg/100 g dry tissue.

Rank	Species	Accession or cultivar	Source	Total betacyanin content (mg/100 g DT)	% Betacyanin composition					*λ*_max_	CIELAB color parameters				
					Amaranthin	Isoamaranthin	Betanin	Isobetanin	Celosianin II		L*	a*	b*	c*	h*
1	*A. cruentus*	PI 566897	USDA-ARS	478.8 ± 52.0_a_	87.3 ± 5.5_d–i_	9.8 ± 4.3_c–g_	1.5 ± 0.2_c–e_	0.1 ± 0.1_b_	1.3 ± 1.3_ab_	536.8 ± 0.7_a–d_	65.5 ± 0.4_a_	62.2 ± 0.5_ab_	−25.2 ± 1.3_gh_	67.1 ± 0.6_a_	−22.0 ± 1.0_gh_
2	*A. cruentus*	Hopi Red Dye	Seeds of Change	385.4 ± 189.9_ab_	88.1 ± 6.2_c–i_	10.8 ± 6.1_c–g_	0.4 ± 0.3_c–e_	0.1 ± 0.1_b_	0.7 ± 0.3_ab_	535.9 ± 0.3_a–d_	64.7 ± 0.3_a–d_	59.7 ± 1.0_a–e_	−20.4 ± 1.6_d–h_	63.1 ± 1.5_a–e_	−18.9 ± 1.1_d–h_
3	*A. cruentus*	PI 674259	USDA-ARS	374.9 ± 45.2_ab_	91.4 ± 4.5_a–f_	7.5 ± 4.2_d–j_	0.5 ± 0.4_c–e_	0.1 ± 0.2_b_	0.5 ± 0.4_ab_	534.1 ± 1.2_ab_	65.4 ± 0.5_ab_	61.5 ± 1.1_a–c_	−23.0 ± 2.1_f–h_	65.6 ± 1.7_ab_	−20.5 ± 1.5_f–h_
4	*A. cruentus*	Hopi Red Dye MU 560	West Coast Seeds	330.0 ± 118.7_bc_	88.0 ± 6.6_c–i_	11.0 ± 6.3_c–f_	0.3 ± 0.2_c–e_	0.1 ± 0.1_b_	0.6 ± 0.5_ab_	534.0 ± 1.0_a–c_	64.8 ± 0.3_a–d_	60.5 ± 1.0_a–d_	−22.3 ± 2.2_e–h_	64.5 ± 1.7_a–d_	−20.2 ± 1.5_e–h_
5	*A. cruentus*	Polish	USDA-ARS	328.9 ± 28.1_b–c_	89.6 ± 3.8_b–h_	8.8 ± 3.7_d–h_	0.7 ± 0.5_c–e_	0.1 ± 0.1_b_	0.8 ± 0.6_ab_	535.8 ± 0.2_a–f_	65.1 ± 0.5_a–c_	60.6 ± 1.4_a–d_	−21.8 ± 2.0_e–h_	64.4 ± 1.9_a–d_	−19.7 ± 1.3_e–h_
6	*A. cruentus*	Ames 5384	USDA-ARS	322.2 ± 43.2_bc_	89.7 ± 6.2_b–h_	8.5 ± 4.9_d–h_	0.4 ± 0.4_c–e_	0.1 ± 0.2_b_	1.3 ± 1.5_ab_	536.5 ± 1.9_a–d_	65.2 ± 0.6_a–c_	61.6 ± 2.4_a–c_	−22.1 ± 0.9_e–h_	65.4 ± 2.4_a–c_	−19.8 ± 0.8_e–h_
7	*A. cruentus*	Hopi Red Dye	Baker Creek Seeds	283.0 ± 81.0_b–d_	87.5 ± 7.5_c–i_	11.9 ± 6.8_b–e_	0.3 ± 0.4_c–e_	0.1 ± 0.1_b_	0.2 ± 0.4_ab_	536.3 ± 2.6_a–f_	64.9 ± 0.2_a–d_	59.8 ± 0.5_a–d_	−19.6 ± 1.5_d–h_	62.9 ± 0.9_a–e_	−18.1 ± 1.2_c–h_
8	*A. sp*	PI 689689	USDA-ARS	270.9 ± 67.7_b–e_	90.5 ± 3.3_a–g_	8.4 ± 3.2_d–i_	1.1 ± 0.7_c–e_	0.1 ± 0.1_b_	0.1 ± 0.1_b_	536.4 ± 0.6_a–f_	65.7 ± 0.3_a_	62.3 ± 1.0_a_	−26.3 ± 1.6_h_	67.7 ± 1.5_a_	−22.8 ± 1.0_h_
9	*A. cruentus*	PI 665286	USDA-ARS	267.0 ± 65.8_b–f_	92.3 ± 3.1_a–f_	6.9 ± 2.8_d–j_	0.4 ± 0.4_c–e_	ND_b_	0.4 ± 0.5_ab_	536.5 ± 0.7_a–d_	65.1 ± 0.3_a–c_	60.3 ± 0.8_a–d_	−21.8 ± 1.7_e–h_	64.1 ± 1.3_a–e_	−19.8 ± 1.2_e–h_
10	*A. cruentus*	PI 511713	USDA-ARS	234.2 ± 54.6_c–f_	90.9 ± 4.8_a–g_	7.4 ± 4.0_d–j_	0.8 ± 1.0_c–e_	ND_b_	0.9 ± 0.6_ab_	536.3 ± 0.9_a–f_	64.7 ± 0.3_a–d_	58.9 ± 0.5_a–h_	−16.2 ± 1.8_b–h_	61.1 ± 0.7_a–g_	−15.3 ± 1.7_b–h_
11	*A. cruentus*	PI 511714	USDA-ARS	231.7 ± 53.9_c–f_	91.7 ± 3.5_a–f_	7.1 ± 3.2_d–j_	0.3 ± 0.3_de_	0.1 ± 0.1_b_	0.8 ± 1.2_ab_	536.3 ± 0.8_a–f_	64.7 ± 0.3_a–d_	59.0 ± 0.9_a–h_	−16.2 ± 3.1_b–h_	61.2 ± 1.6_a–g_	−15.3 ± 2.6_b–h_
12	*A. sp*	Four Star Explorers Mix	Wild Garden Seed	200.4 ± 95.0_c–g_	87.0 ± 4.9_e–i_	10.5 ± 4.2_c–g_	0.7 ± 0.6_c–e_	0.1 ± 0.1_b_	1.7 ± 1.1_ab_	536.2 ± 0.5_a–e_	64.7 ± 0.3_a–d_	59.4 ± 1.4_a–f_	−19.4 ± 3.7_d–h_	62.6 ± 2.4_a–e_	−18.0 ± 3.0_c–h_
13	*A. cruentus*	Velvet Curtains	Sunshine Flower Seeds	198.0 ± 104.0_c–g_	88.2 ± 4.6_c–i_	9.8 ± 4.2_c–g_	0.5 ± 0.5_c–e_	ND_b_	1.5 ± 1.0_ab_	536.5 ± 1.3_a–f_	64.6 ± 0.4_a–e_	59.1 ± 1.8_a–g_	−18.9 ± 3.7_c–h_	62.1 ± 2.8_a–f_	−17.6 ± 2.9_c–h_
14	*A. cruentus*	PI 633592	USDA-ARS	173.7 ± 75.0_d–h_	86.0 ± 7.7_f–j_	12.6 ± 6.8_b–e_	0.9 ± 0.9_c–e_	0.1 ± 0.1_b_	0.4 ± 0.6_ab_	536.8 ± 1.1_a–f_	64.3 ± 0.2_a–f_	57.5 ± 0.6_a–j_	−13.9 ± 3.4_b–h_	59.2 ± 1.3_b–h_	−13.6 ± 3.0_b–h_
15	*A.tricolor*	PI 277269	USDA-ARS	173.0 ± 15.3_d–i_	81.3 ± 5.1_g–j_	14.8 ± 4.3_a–d_	3.6 ± 1.0_a–e_	0.3 ± 0.4_ab_	ND_b_	535.7 ± 0.1_a–f_	63.7 ± 0.4_a–f_	57.4 ± 1.1_b–j_	−14.0 ± 2.2_b–h_	59.1 ± 1.6_b–h_	−13.7 ± 1.8_b–h_
16	*A.tricolor*	PI 419057	USDA-ARS	156.7 ± 63.8_d–i_	83.8 ± 6.6_f–j_	11.8 ± 4.4_b–e_	4.0 ± 1.9_a–d_	0.4 ± 0.5_ab_	ND_b_	536.3 ± 0.6_a–f_	64.9 ± 1.0_a–d_	59.7 ± 1.9_a–e_	−19.0 ± 5.1_c–h_	62.7 ± 3.3_a–e_	−17.5 ± 3.9_c–h_
17	*A.tricolor*	PI 603897	USDA-ARS	144.3 ± 26.7_e–j_	88.0 ± 6.6_c–i_	7.6 ± 3.9_d–j_	4.1 ± 2.6_a–c_	0.2 ± 0.3_b_	0.1 ± 0.1_b_	535.5 ± 0.9_a–f_	63.9 ± 0.7_a–f_	57.6 ± 1.5_a–j_	−13.9 ± 1.9_b–h_	59.3 ± 1.8_b–h_	−13.5 ± 1.6_b–h_
18	*A. cruentus*	Burgundy	Seeds of Change	144.2 ± 51.2_e–j_	87.5 ± 2.8_d–i_	10.5 ± 3.5_c–g_	0.3 ± 0.4_de_	ND_b_	1.7 ± 0.8_ab_	535.8 ± 0.1_a–f_	64.2 ± 0.5_a–f_	57.1 ± 1.5_c–k_	−14.3 ± 3.8_b–h_	58.9 ± 2.3_b–h_	−14.0 ± 3.2_b–h_
19	*A. quitensis*	PI 490710	USDA-ARS	143.7 ± 41.3_e–j_	92.4 ± 3.6_a–f_	6.0 ± 3.1_e–j_	1.4 ± 0.6_c–e_	ND_b_	0.1 ± 0.1_b_	535.8 ± 0.1_a_	64.4 ± 0.3_a–f_	58.5 ± 1.5_a–i_	−20.5 ± 4.8_d–h_	62.1 ± 2.7_a–f_	−19.2 ± 4.0_d–h_
20	*A. cruentus*	Red Spike	Johnny’s Seeds	141.8 ± 65.0_e–j_	86.5 ± 5.7_e–i_	11.1 ± 4.7_c–f_	0.2 ± 0.3_e_	0.5 ± 0.9_ab_	1.7 ± 0.8_ab_	535.6 ± 0.4_a–f_	64.2 ± 0.8_a–f_	57.2 ± 1.8_c–k_	−14.6 ± 1.9_b–h_	59.0 ± 2.2_b–h_	−14.2 ± 1.4_b–h_
21	*A. cruentus*	Burgundy Grain MU551	West Coast Seeds	138.8 ± 63.9_e–k_	87.4 ± 6.3_d–i_	11.2 ± 5.7_c–f_	0.2 ± 0.4_e_	ND_b_	1.2 ± 0.9_ab_	536.2 ± 0.5_a–d_	63.9 ± 0.5_a–f_	56.3 ± 1.5_d–k_	−12.0 ± 3.6_b–g_	57.7 ± 2.2_e–h_	−11.9 ± 3.2_b–h_
22	*A. cruentus*	Garnet Red	Johnny’s Seeds	138.6 ± 41.7_e–_	86.9 ± 5.7_e–i_	11.1 ± 5.2_c–f_	0.3 ± 0.5_de_	0.1 ± 0.1_b_	1.7 ± 0.8_ab_	535.9 ± 0.1_a–f_	64.3 ± 0.2_a–f_	57.3 ± 0.6_b–k_	−14.5 ± 2.6_b–h_	59.2 ± 1.2_b–h_	−14.2 ± 2.4_b–h_
23	*A. tricolor*	PI667171	USDA-ARS	126.6 ± 29.1_f–k_	84.6 ± 7.7_f–j_	12.3 ± 6.0_b–e_	2.7 ± 1.4_a–e_	0.4 ± 0.5_ab_	ND_b_	534.5 ± 1.1_d–f_	62.8 ± 0.7_c–f_	54.5 ± 1.3_f–k_	−8.0 ± 4.2_a–d_	55.2 ± 1.8_gh_	−8.2 ± 4.2_a–f_
24	*A. tricolor*	Ames 2214	USDA-ARS	124.5 ± 43.7_f–k_	84.6 ± 5.8_f–j_	8.8 ± 3.4_d–h_	6.4 ± 2.7_a_	0.1 ± 0.2_b_	0.1 ± 0.3_ab_	535.8 ± 0.1_e–f_	62.5 ± 0.5_d–f_	54.8 ± 0.9_e–k_	−9.5 ± 3.4_a–e_	55.7 ± 1.5_f–h_	−9.8 ± 3.2_b–g_
25	*A. cruentus*	PI 647848	USDA-ARS	120.0 ± 81.6_f–k_	85.3 ± 5.6_f–j_	11.7 ± 6.2_c–e_	1.9 ± 3.4_b–e_	0.2 ± 0.3_b_	0.9 ± 1.1_ab_	536.1 ± 0.5_a–d_	64.4 ± 1.0_a–f_	56.7 ± 3.7_c–k_	−10.7 ± 6.8_a–f_	58.0 ± 5.0_d–h_	−10.3 ± 5.8_b–h_
26	*A. tricolor*	Ames 25,153	USDA-ARS	117.1 ± 78.3_f–k_	83.4 ± 5.9_f–j_	13.0 ± 3.9_b–e_	3.5 ± 1.9_a–e_	0.2 ± 0.3_b_	ND_b_	535.4 ± 0.6_b–f_	63.1 ± 1.4_b–f_	56.3 ± 3.0_d–k_	−12.3 ± 7.3_b–g_	57.9 ± 4.3_d–h_	−12.0 ± 6.6_b–h_
27	*A. tricolor*	PI 608761	USDA-ARS	114.3 ± 29.6_f–k_	84.9 ± 7.1_f–j_	11.2 ± 5.1_c–f_	3.6 ± 1.8_a–e_	0.2 ± 0.3_b_	ND_b_	537.1 ± 1.5_a–f_	62.2 ± 1.2_f_	54.1 ± 0.8_h–k_	−7.2 ± 3.8_a–d_	54.7 ± 1.3_gh_	−7.5 ± 3.9_a–e_
28	*A. tricolor*	PI 603898	USDA-ARS	112.5 ± 32.7_f–k_	86.2 ± 1.6_f–j_	10.2 ± 1.5_c–g_	3.5 ± 2.4_a–e_	0.1 ± 0.1_b_	ND_b_	536.0 ± 0.1_f_	62.5 ± 0.9_d–f_	54.2 ± 2.2_g–k_	−7.5 ± 6.7_a–d_	55.0 ± 2.9_gh_	−7.6 ± 6.7_a–f_
29	*A. blitum*	PI 606282	USDA-ARS	110.9 ± 71.0_f–k_	86.0 ± 6.3_f–j_	13.4 ± 6.6_b–e_	0.3 ± 0.4_c–e_	0.1 ± 0.2_b_	0.2 ± 0.4_ab_	533.5 ± 0.5_a–d_	62.0 ± 3.3_f_	57.2 ± 3.5_c–k_	−7.8 ± 14.2_a–d_	58.9 ± 5.3_c-h_	−6.9 ± 13.3_a–d_
30	*A. cruentus*	Black Leaved	Seed Savers Exchange	102.0 ± 28.6_f–k_	87.1 ± 4.1_c–i_	12.1 ± 3.4_c–g_	TRACE_c–e_	ND_b_	0.8 ± 0.7_ab_	534.6 ± 1.0_a–f_	64.5 ± 0.6_a–f_	57.3 ± 1.1_a–k_	−13.8 ± 0.1_b–h_	58.9 ± 1.1_b–h_	−13.6 ± 0.3_b–h_
31	*A. tricolor*	Molten Fire	Livingston Seed	98.7 ± 52.9_f-k_	83.2 ± 7.1_f–j_	9.2 ± 3.1_d–j_	5.9 ± 3.4_ab_	1 ± 1_a_	0.7 ± 0.7_ab_	534.2 ± 0.5_b–f_	63.5 ± 1.4_a–f_	56.2 ± 4.1_c–k_	−6.0 ± 8.1_a–d_	56.9 ± 5.0_e–h_	−5.6 ± 7.6_a–e_
32	*A. tricolor*	PI 674261	USDA-ARS	90.2 ± 27_g–k_	85.9 ± 6.2_f–j_	11.6 ± 5.6_c–e_	2.5 ± 0.9_b–e_	TRACE_b_	ND_b_	534.8 ± 1.2_a–f_	62.2 ± 0.7_ef_	52.7 ± 1.8_jk_	−3.8 ± 7.1_ab_	53.2 ± 2.3_h_	−3.9 ± 7.5_ab_
33	*A. tricolor*	PI 419121	USDA-ARS	82.6 ± 58.2_g–k_	80.5 ± 7.8_h–j_	17.6 ± 6.1_a–c_	1.6 ± 1.8_c–e_	0.2 ± 0.5_b_	ND_b_	535.5 ± 1.6_a–d_	62.3 ± 1.8_ef_	52.4 ± 4.9_k_	2.7 ± 15.7_a_	54.1 ± 5.9_h_	4.1 ± 15.7_a_
34	*A. graecizans*	PI 608661	USDA-ARS	73.5 ± 32.2_g–k_	88.4 ± 8_c–i_	10.9 ± 8.3_c–g_	0.7 ± 0.5_c–e_	ND_b_	ND_b_	533.6 ± 0.5_c-f_	62.3 ± 1.7_ef_	53.9 ± 3.6_i–k_	−5.8 ± 10.5_a–c_	54.9 ± 4.6_gh_	−5.5 ± 10.6_a–c_
35	*A. tricolor*	Red Leaf	West Coast Seeds	67.1 ± 34.5_g–k_	76.8 ± 3_j_	22.3 ± 3.3_a_	0.9 ± 0.6_c–e_	TRACE_b_	ND_b_	_–_	_–_	_–_	_–_	_–_	_–_
36	*A. tricolor*	Ames 2,132	USDA-ARS	57 ± 19.5_h–k_	89 ± 5.2_c–h_	9.5 ± 4.6_c–g_	1.4 ± 1.1_c–e_	ND_b_	ND_b_	_–_	_–_	_–_	_–_	_–_	_–_
37	*A. tricolor*	PI 477918	USDA-ARS	50.5 ± 34.5_g–k_	77.0 ± 8.6_ij_	21.9 ± 8.7_ab_	1.1 ± 0.1_c–e_	ND_b_	TRACE_ab_	_–_	_–_	_–_	_–_	_–_	_–_
38	*A. quitensis*	PI 490712	USDA-ARS	46.6 ± 33.9_h–k_	90.8 ± 3.8_a–g_	7.6 ± 3.4_d–j_	1.3 ± 0.9_c–e_	0.1 ± 0.2_b_	0.2 ± 0.3_ab_	-	_––_	_–_	_–_	_–_	-
39	*A. cruentus*	PI 451711	USDA-ARS	46.6 ± 13.8_h–k_	89.7 ± 7.9_b–h_	8.2 ± 7.0_d–i_	0.3 ± 0.6_de_	ND_b_	1.8 ± 1.4_a_	_–_	_–_	_–_	_–_	_–_	_–_
40	*A. tricolor*	Ames 2,223	USDA-ARS	34.6 ± 18.9_h–k_	89.2 ± 3.9_a–h_	9.2 ± 3.9_d–j_	1.6 ± 0.4_b–e_	ND_b_	TRACE_ab_	_–_	_–_	_–_	_–_	_–_	_–_
41	*A. dubius*	PI 605352	USDA-ARS	30.5 ± 31.3_h–k_	99.0 ± 1.8_ab_	1.0 ± 1.8_h–j_	TRACE_c–e_	ND_b_	TRACE_ab_	_–_	_–_	_–_	_–_	_–_	_–_
42	*A. cruentus*	Big Red	Seed Savers Exchange	19.7 ± 3.2_h–k_	92.9 ± 2.3_a–f_	5.7 ± 2.3_e–j_	TRACE_c–e_	ND_b_	1.4 ± 1.5_ab_	_–_	_–_	_–_	_–_	_–_	_–_
43	*A. tricolor*	Aurora Yellow	Baker Creek Seeds	15.2 ± 11.7_i–k_	97.1 ± 5_a–c_	TRACE_j_	2.9 ± 5.0_a–e_	ND_b_	TRACE_ab_	_–_	_–_	_–_	_–_	_–_	_–_
44	*A. cruentus*	Ames 2234	USDA-ARS	13.3 ± 7.4_jk_	96.9 ± 6.3_a–d_	3.1 ± 6.3_e–j_	ND_e_	ND_b_	ND_b_	_–_	_–_	_–_	_–_	_–_	_–_
45	*A. cruentus*	Dreadlocks	Wild Garden Seed	12.8 ± 7_jk_	TRACE_a_	TRACE_ij_	ND_e_	ND_b_	ND_b_	_–_	_–_	_–_	_–_	_–_	_–_
46	*A. caudatus*	PI 553073	USDA-ARS	11.1 ± 4_jk_	TRACE_ab_	TRACE_h–j_	ND_e_	ND_b_	ND_b_	_–_	_–_	_–_	_–_	_–_	_–_
47	*A. cruentus*	Hot Biscuits	Wild Garden Seed	6.4 ± 1.4_k_	TRACE_a_	TRACE_ij_	ND_e_	ND_b_	ND_b_	_–_	_–_	_–_	_–_	_–_	_–_
48	*A. hypochondriacus*	PI 558499	USDA-ARS	4.7 ± 2.4_k_	TRACE_a–e_	TRACE_g–j_	TRACE_c–e_	ND_b_	TRACE_ab_	_–_	_–_	_–_	_–_	_–_	_–_

Data are presented as the mean of all greenhouse replications (*n* = 4 or 3) ±standard deviation. Tukey pairwise comparisons were made to determine significant differences between genotypes, indicated with subscript letters by column. Means that do not share a letter have a statistically significant difference (*α* = 0.05). Spectral data for accessions with low pigmentation are omitted due to the lack of a distinct maximum peak around 536 nm at the concentration analyzed.

Plants were grown in the greenhouse facilities at the University of Illinois at Urbana-Champaign in four replicates, planted in February 2018, August 2018, September 2018, and May 2019. In each replicate, five individual plants were grown for each accession. Seeds were sown directly into 5-inch pots filled with Sunshine® LC1 (Sun Gro Horticulture, Vancouver, BC, Canada) professional soil mix and thinned to 1 plant per pot upon germination. Plants were grown under a 31/23°C, 15/9 h day/night temperature regime with high-pressure sodium (HPS) lighting provided from 06:00 to 21:00 h if light intensities fell below 900 watts/m^2^. Plants were manually watered daily and fertilized with an organic 4–1-1 fertilizer (Pennington Seed, CA) weekly starting at week 2. All above-ground biomass was harvested 7 weeks after planting and immediately frozen at-20°C. This frozen plant material was then lyophilized, ground into a fine powder, and stored at-20°C until analysis.

### Method optimization for extraction of betalains

Pigments were extracted from lyophilized powdered material in 50 ml polypropylene centrifuge tubes using 40 ml of deionized water. Response surface methodology (RSM) was used to optimize extraction variables so that total betacyanin yield from a single extract was maximized. This method is commonly used to optimize complex biotechnological processes in food research and consists of plotting multiple independent factors at different levels (e.g., extraction times of 10, 20, and 30 min) in a three-dimensional space so that their interaction with a response variable can be precisely monitored as a response surface plane (Box and Wilson, 1951). This surface can be interpreted to yield the best conditions for the desired response. In this study, a Box–Behnken RSM design was used to optimize extraction time (10 min, **20 min**, 30 min), temperature (**30°C**, 40°C, 50°C) and plant mass (**0.15 g**, 0.25 g, 0.35 g) so that betacyanin pigment yields were maximized. Bolded conditions were selected after optimization trials and used for all experimental samples. Optimization trials were conducted on five randomly selected amaranth accessions, coded as a categorical variable during analysis, to better represent the extractability of the broad range of accessions surveyed. After extraction, tubes were centrifuged at a relative centrifugal force (RCF) of 3,000× *g* for 5 min. Supernatants were then filtered through a 30 μm CellTrics cell strainer (Sysmex, IL, United States) followed by a 0.45 μm hydrophilic PFTE syringe filter (Thermo Fisher Scientific, Indianapolis, United States) and chilled on ice until analysis. To limit protein precipitation under acidic HPLC conditions, samples for HPLC analysis were acidified with a small amount of formic acid after straining (1% v/v final concentration), briefly vortexed, and centrifuged in a 2 ml tube for 10 min at 21100 RCF prior to syringe filtration. This additional step was used rather than simply acidifying the starting extraction solvent to avoid the accelerated degradation observed for betalains at high temperatures and low pH values (Pátkai and Barta, 1996).

### UV–Vis spectroscopy and colorimetric analyses

Spectral readings for ultraviolet–visible (UV–Vis) well plate assays were collected using a Synergy 2 multi-well plate reader (Biotek, Winooski, VT, USA). A final volume of 200 μl was added to each well and any bubbles were removed by blowing ethanol vapor from a squeeze bottle across the sample(s). Data were corrected to a pathlength of 1 cm by analyzing select samples for each extraction system in a reference 1 cm pathlength cuvette using a UV–Vis spectrophotometer (Shimadzu-1800, Japan). These cuvette-derived data were divided by data from the plate reader to obtain the correction factor for each extraction system.

Extract color parameters were calculated using the CIE (Commission internationale de l’éclairage) L^*^ a^*^ b^*^ (CIELAB) color space. Parameters were calculated with *ColorBySpectra* software (Farr and Giusti, 2019) using 380–780 nm spectra in 1 nm intervals normalized to A_max_ = 1 using the following settings: 10° observer angle, illuminant D65, pathlength 1 cm. Chroma (C^*^) and hue angle (h^*^) were calculated according to the equations below:


$$C^{*}=\sqrt{{\left(a^{*}\right)}^{2}+{\left(b^{*}\right)}^{2}}$$

$$h^{*}=\tan^{-1}\left(\frac{b^{*}}{a^{*}}\right)$$

where a^*^ and b^*^ correspond to the CIELAB color space values in which L^*^ measures lightness, a^*^ redness/greenness, and b^*^ blueness/yellowness. Samples were buffered with a 0.1 M pH 6.5 phosphate buffer prior to analysis. For each accession, samples were analyzed independently from three separate extractions prior to being averaged.

### Quantification of betacyanins

Total betacyanin concentrations were determined as described by Cai et al. (1998a) using a well-plate spectrophotometer with manual pathlength correction as previously mentioned (Section “UV–Vis spectroscopy and colorimetric analyses”). All samples were diluted with 0.1 M pH 6.5 phosphate buffer before analysis. The following equation was used:


Pigment Content(mg100gdrymass)=A536×MW×DF×V×100ε×M×L

In the equation, *A*_536_ = absorbance at a 536 nm, MW = molecular weight, DF = dilution factor, *V* = extraction volume (ml), *ε* = molar coefficient, *M* = mass (g) of material being extracted, and *L* = pathlength (cm). Coefficients for amaranthin (*ε* = 56,600 l/mol · cm; molecular weight = 726) were used (Piattelli et al., 1969). Samples were extracted and read in triplicate.

### HPLC analyses of betalains

Betalain composition analyses were carried out as described by Cai et al. (2001a). An HPLC-system (Hitachi High Technologies America, Inc., Schaumburg, IL), equipped with a L-7200 autosampler, D-7000 interface module, L-7100 pump, and a L-7455 diode array detector was used for pigment analyses. Separation was achieved using a LUNA Phenomenex C_18_ reversed-phase column (5 μm, 250 × 4.6 mm, Torrance, CA) and a SecurityGuard C_18_ guard column (Phenomenex; Torrance, CA). The mobile phase used 1.5% (v/v) phosphoric acid as solvent A and 1.5% phosphoric acid, 20% acetic acid, and 25% acetonitrile for solvent B. A 30-min linear gradient from 10 to 55% solvent B in solvent A was used, with an added 1-min 100% B wash followed by a 5-min equilibration at 10% B after each sample. An injection volume of 20 μl was used with monitoring at 535 nm. The column was kept at ambient temperature at a constant flow rate of 1 ml/min and samples were injected *via* a 20 μl sample loop. Purified betanin (Millipore-Sigma; St. Louis, MO) was used as a standard for compound identification with each set of samples analyzed, and identified compounds were quantified as a percentage of the total betacyanin content determined previously using a UV–Vis spectrophotometer. Spectral DAD data were simultaneously collected between 250 and 600 nm.

### LC-ESI-MS–MS analysis

Liquid chromatography-electrospray ionization-mass spectrometry (LC-ESI-MS–MS) was performed on selected samples with diverse pigment profiles (i.e., PI 566897, ‘Four Star Explorers Mix’, ‘Hot Biscuits’) to confirm the identity of peaks observed using HPLC. Analyses were performed at the School of Chemical Sciences Mass Spectrometry Lab at the University of Illinois with a Poroshell 120 SB-C18 (100 mm × 2.1 mm; 2.7 μm; Agilent, CA) at ambient temperature using 1% formic acid in water as solvent A and 1% formic acid in acetonitrile as solvent B. A gradient of 100% solvent A to 80% solvent B over 10 min with a flow rate of 0.7 ml/min was used. Mass spectra were acquired in positive mode scanning in a mass to charge ratio (m/z) range of 100–1,000 at a cone voltage of 35 V. Spectral data were collected at 535 nm. Compound identities were confirmed based on published literature of MS and MS–MS measurements and standard compounds.

### Statistical analyses

Results in Table 1 are expressed as the mean ± standard deviation of four independent greenhouse trials or replicates. For each trial, five individual plants per accession were bulked together at harvest. In one of the greenhouse trials, some unknown factor affected the germination and growth of plants for seven accessions, so these are represented by three replications in Table 1. Otherwise, germination and growth for the different accessions were not issues during the trials. Significant differences were calculated by one-way ANOVA (H_0_: total betacyanin content is the same across all tested accessions) using a generalized linear model with replication as a blocking factor, followed by Tukey’s HSD procedure to perform pairwise mean comparisons (α = 0.05). Response surface methodology experiments for optimization of betalain extractions were analyzed using Minitab 19 (MINITAB Inc., PA). Statistical correlations were made using the non-parametric Spearman rank correlation test.

## Results

### Optimization of extraction methods

Harvested plant material was lyophilized to facilitate homogenization of tissue *via* grinding and to maximize stability of pigments in storage prior to analysis. Water was chosen as the solvent for betalain extractions to maximize the direct applications of our results to the food industry. FDA guidelines (CFR Title 21 73.260) state that all natural colorants labeled as ‘vegetable juice’ must either be expressed from fresh tissue or extracted from dry tissue with a simple water infusion—any specialized extraction or purification protocol would likely require a significant investment of time and money to gain explicit regulatory approval in the US and beyond.

The temperature, time, and mass of extraction material for betalain extractions were optimized using response surface methodology with a Box–Behnken design. Example contour plots showing the effects of extraction parameters on betacyanin content are shown in Supplementary Figure S1. All three variables, along with accession (i.e., labeled genotype in the figure), were found to significantly affect betalain content (*p* < 0.01) with an adjusted *R*^2^ value of 83.1%. The interactions between time^*^temperature, temperature^*^mass, and temperature^*^accession were also significant (*p* < 0.05). Accession or genotype was the most influential factor, followed by mass, temperature^*^accession, time, time^*^temperature, temperature, and lastly temperature^*^mass. Using the optimization tool to maximize betalain content, the optimal parameters selected for further experimental analyses were a temperature of 30°C, incubation for 20 min, and the use of 0.15 g of ground lyophilized tissue in 40 ml of deionized water. The efficiency of this first extraction was found to be 96.2% (SD = 0.0062), which was determined from 10 separate successive extractions of the same starting material.

### Betalain yield and composition

Table 1 presents mean values of betacyanin content, composition, and spectral qualities for all forty-eight accessions surveyed. Those accessions with the highest betacyanin yield (BCY) belonged to *A. cruentus* and suspected hybrids thereof. Accession PI 566897 (*A. cruentus*) had the highest betacyanin yield at 478.8 mg BCY/100 g DT when averaged across all four replicates. The single highest betacyanin yield within a single replicate was in *A. cruentus* ‘Hopi Red Dye’ (Seeds of Change, CA) with 655.6 mg betacyanins (BCY)/100 g dry tissue (DT; Supplementary Table S1). No betaxanthins were detected spectrophotometrically in any of the accessions, but this was not altogether unexpected given this subcategory of betalains are typically minor components localized in flowers and fruits of betalain-producing plants.

**Figure 1:**
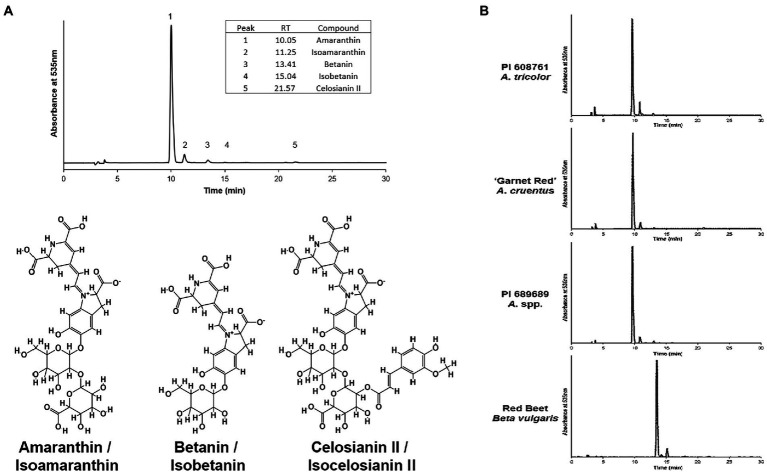
**(A)** Representative HPLC chromatogram of an *A. cruentus* extract (PI 566897) using the analysis method described by Cai et al. (2001a). Example normalized chromatograms of PI 608761 (*A. tricolor*), ‘Garnet Red’ (*A. cruentus*), PI 689689 (*A. cruentus*), and an extract of store-bought red beet (*Beta vulgaris*) are shown in **(B)** to emphasize the relatively minor scale of compositional differences between amaranth cultivars and, in contrast, their substantial differences in comparison to betanin-dominant beet extracts.

**Figure 2:**
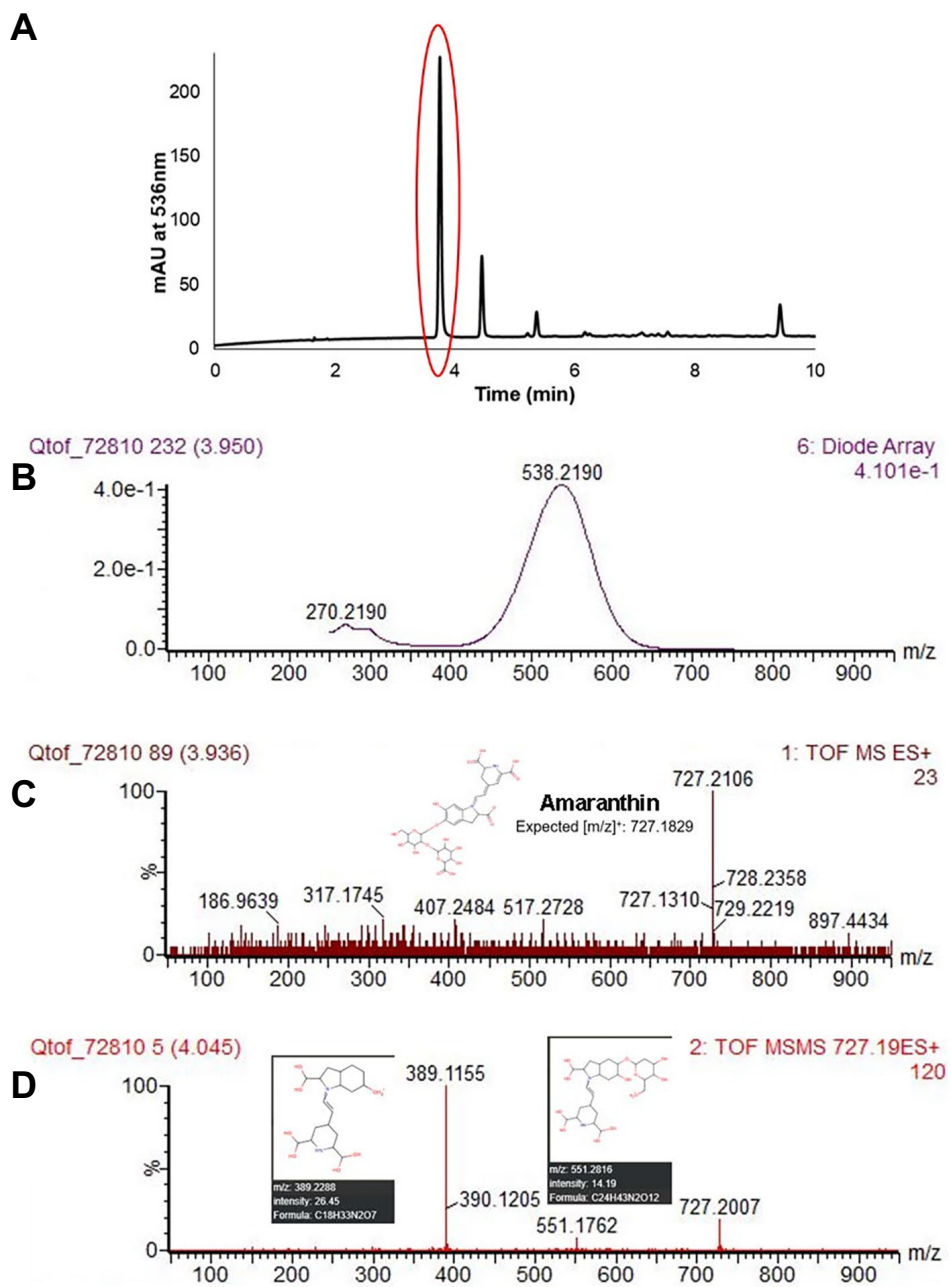
HPLC chromatogram of *A. cruentus* ‘Hopi Red Dye’ (Seeds of Change) using the HPLC-MS–MS protocol described in section “LC-ESI-MS-MS analysis” **(A)**. Representative spectral DAD data **(B)**, MS **(C)** and MS–MS **(D)** results at 3.95 min are displayed for the first and most abundant peak in **(A)**, amaranthin. Fragmented ions in **(D)** matched [m/z] predicted by the Human Metabolome LC–MS database (Wishart et al., 2018). Lower limits of detection and quantitation were estimated empirically by analysis of small concentrations of betanin standard.

Five different betacyanin compounds were identified from the water extracts and summarized as percentages of total betacyanin concentration (Table 1). Peak and compound identities are summarized in Figure 1A with example chromatograms from different amaranth accessions and beet shown in Figure 1B. Amaranthin was the major betacyanin compound present in all accessions surveyed, composing an average of 88.6% of the total betacyanin content. Minor betacyanins included isoamaranthin (*x̄* = 9.4%), betanin (*x̄* = 1.4%), isobetanin (*x̄* = 0.1%), and celosianin II (*x̄* = 0.5%). An example of the HPLC-MS–MS method used is shown in Figure 2, confirming the identity of amaranthin.

### Color measurements of betalain extracts

Color parameters were measured from the same samples used for determining total betalain content, buffered to a pH of 6.5 and normalized to *A*_max_ = 1 to allow for direct comparisons at equal tinctorial strengths. Only lines with considerable betacyanin concentrations (>70 mg/100 g DT) were analyzed (Table 1). The visual color of these extracts ranged from magenta to pink. The lack of betaxanthins was visually apparent once extracts were filtered; unlike most raw beet extracts that appear true red, betaxanthin-free amaranth extracts appear as a bluer and highly saturated magenta color (Supplementary Figure S2). One USDA accession of *A. cruentus* (PI 689689) had a uniquely low b^*^ value, with a vivid magenta appearance (Supplementary Figure S2A, #1). The λ_max_ values of pigmented accessions were relatively constant, with an average value of 535.7 nm (Supplementary Table S1). Accessions with lower BCY values, however, generally had lower λmax values suggesting that a larger proportion of partially degraded betacyanins were present. Significant correlations were observed between color values and HPLC data, with higher proportions of amaranthin negatively correlating with hue (*r* = −0.4756, *p* < 0.01) and positively with chroma (*r* = 0.46, p < 0.01) to yielding bluer and more saturated colors with increasing proportions of amaranthin. These correlations, however, appear to be confounded by total betacyanin content— regression analysis (not shown) indicated that betacyanin composition did not significantly affect any color parameters when total betacyanin content was accounted for. Given this information, the variability seen in b^*^ is likely due to the presence of unquantified dehydrogenated or decarboxylated betacyanins, betalamic acid, and other non-betalain compounds selectively absorbing blue light.

## Discussion

### Phenotypic characterization of genotypes

**Figure 3:**
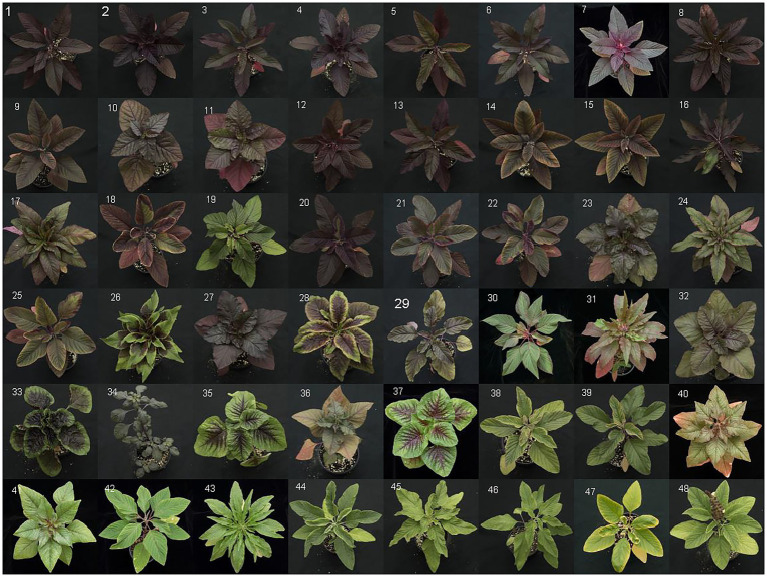
Aerial photographs of all accessions examined in this study. Photos are arranged by average total betacyanin yield in descending order. Accession numbers, cultivar names, and seed sources are listed in Table 1 with the corresponding numbers listed on each picture.

**Figure 4:**
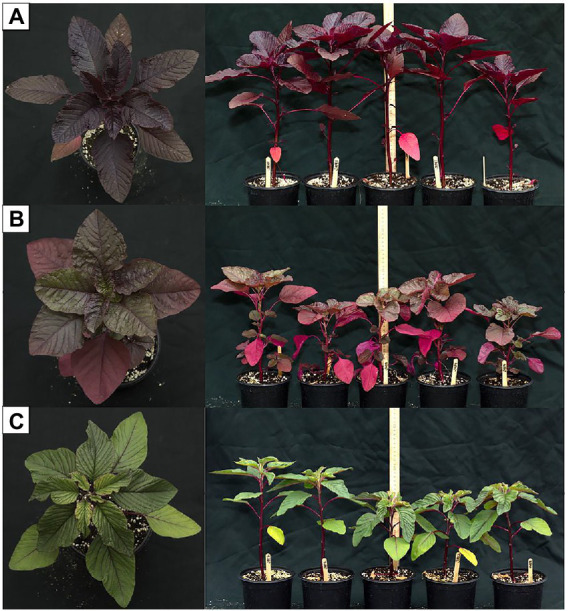
Aerial and profile photographs of **(A)** *A. cruentus* ‘Hopi Red Dye’ (Seeds of Change), **(B)** *A. tricolor* PI 277269, and **(C)** *A. quitensis* PI 490710 as examples of variation in morphology and pigment localization among species and cultivars in our study. Betacyanin pigments were visually apparent when present.

Sampled accessions of *Amaranthus cruentus* generally showed the most visually intense pigmentation in leaves and stems among all amaranth types examined (Figures 3, 4A). *Amaranth cruentus* includes many landraces, cultivars, and commercial varieties with distinct pigment patterns ranging from completely green, or no discernible betalains at any growth stage, to deeply pigmented stems and leaves at all vegetative growth stages. While floral pigmentation was not the focus of our study, we should point out that some *A. cruentus* genotypes, or hybrids derived from this species, with non-pigmented leaves and stems produce floral tissues with an array of pigment patterns. Two accessions tested, USDA PI 689689 and ‘Four Star Explorers Mix’ (Wild Garden Seed), were listed to be of hybrid origin, presumably involving *A. cruentus*, and both ranked in the top-third of all tested types based on total betacyanin content (Table 1). Varieties of *A. tricolor* stood out from other amaranth species in phenotypic traits due to their shorter growth habit and generally thicker deltoid or lanceolate leaves (e.g., Figure 4B). Unlike *A. cruentus* and other grain amaranths, *A. tricolor* is predominantly consumed as a leafy vegetable or grown as an ornamental for its characteristic blotches of pigmentation among its leaves (e.g., #26, 28, 33, 35, 37 in Figure 3). By comparison, all individuals of one accession of *A. quitensis* (PI 490710; #19 in Table 1 and Figure 4C), a wild, weedy or semi-cultivated species from South America, displayed a visually distinct hue of pigments in stem tissue and localization of betalains at the leaf margins and veins that was unique compared to all other accessions examined. Interestingly, this accession of *A. quitensis* with mostly green leaves ranked higher in total betalains than several amaranth accessions with visually darker pigmentation patterns (Table 1).

### Factors affecting betalain yield

As previously stated, water was chosen as our extraction solvent in accordance with FDA guidelines. Lyophilization of water extracts maximized stability of pigments prior to analysis as betalains are known to have excellent stability when stored under moisture-free conditions (Cai and Corke, 2001). For solely experimental research of pigments and other compounds that may influence pigmentation, solvents other than water may offer advantages in pigment yields, in extracting less-polar co-pigments (Fathordoobady et al., 2016), or in reducing co-extraction of unwanted proteins (Cai et al., 2001a).

In our analysis, betacyanin concentrations in *A. cruentus* PI 566897 are lower than the highest values previously reported for this accession (127 mg BCY/100 g fresh tissue (FT); Cai et al., 2001a), and similarly in *A. tricolor* PI 419057 (143 mg BCY/100 mg FT; Cai et al., 1998a). In these prior studies, betacyanin values were derived exclusively from leaf tissue, equal to approximately 850 mg BCY/100 g and 950 mg BCY/100 g, respectively, from dry plant material assuming a water content of 85%. Our analysis, which differs in methodological approach and end goals from these prior studies, included stem tissue along with leaf material as total above-ground extracted biomass, so the differences in betacyanin yields are not unexpected. Similar to anthocyanins (Steyn et al., 2002), betalains found in above-ground tissue are often sequestered to epidermal tissues (Supplementary Figures S3A,B); stems are a considerable proportion of total harvested biomass and have a relatively low surface area, therefore lowering betacyanin yield per gram of homogenized tissue. The longer growth period utilized in our study could also lower BCY concentrations on a per-gram basis (Cai et al., 1998a). Pigment concentrations reported in the present study are likely more representative of what would be observed if plants are grown and harvested at an industrial scale, where plants would simply be mowed down rather than selectively harvested for leaf tissue only.

**Figure 5:**
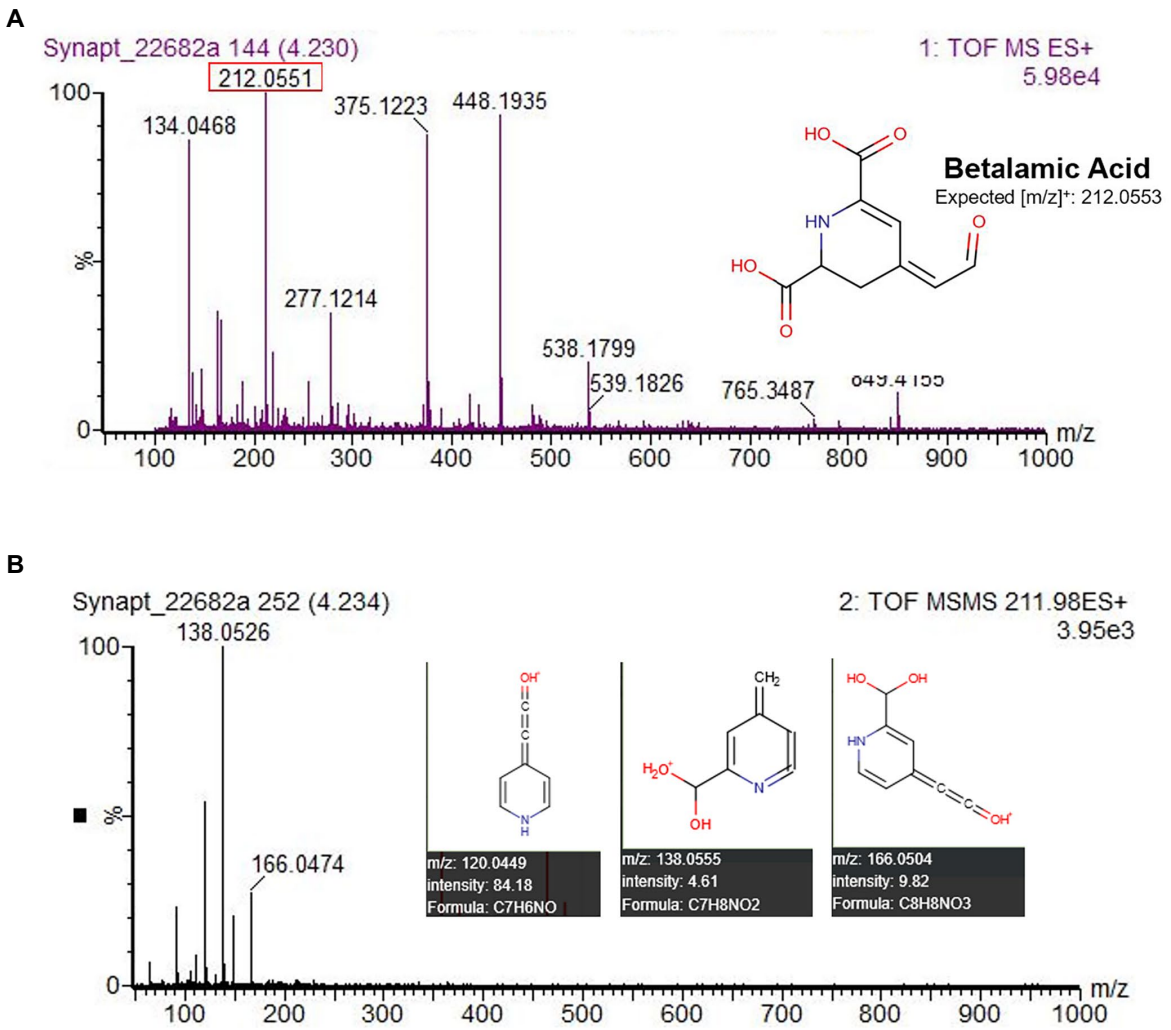
HPLC-MS–MS identification of betalamic acid in *A. cruentus* ‘Hot Biscuits’ (Wild Garden Seed) stem tissue. The protocol was used for analysis with the substitution of solvent A as 100% water and solvent B as 100% acetonitrile. MS–MS data **(B)** matched predicted [m/z] by the Human Metabolome LC–MS database (Wishart et al., 2018), confirming its identity.

All UV–Vis spectra lacked a peak in the 470–490 nm range characteristic of betaxanthins (Supplementary Figure S2). This absence of detectable betaxanthins in leaf and stem material, the focus of our study, agrees with findings from other studies of amaranth (Piattelli, 1976; Cai et al., 1998a; Cai and Corke, 2001). Yellow pigmentation of the accession Ames 25,153 and cultivar ‘Aurora Yellow’, marketed for their variegated leaf coloration with localized yellow sections at maturity, was found in our study to originate from carotenoid pigments (data not shown) most visible in leaf areas devoid of chlorophyll (Supplementary Figure S4). Several studies appear to erroneously identify these carotenoids as betaxanthins due to their co-extraction in alcohol-water solvents and similar UV–Vis spectra that selectively absorb blue light (Khanam and Oba, 2013; Teng et al., 2016; Liu et al., 2019). Unlike carotenoids, betaxanthins can easily be identified by their solubility in water and autofluorescence under blue and UVA illumination (Harris et al., 2012). Very minor concentrations of betalamic acid, a yellow precursor to both betaxanthins and betacyanins, were detected in most accessions and confirmed by LC–MS–MS. The yellow-orange pigmentation of stem and leaf vein tissue of the *A. cruentus* cultivar’ Hot Biscuits’ was found to be attributable to the presence of betalamic acid (Figure 5). Although we did not specifically seek to quantify flavonoids in our extracts, future investigations should consider these compounds as potential co-pigments.

Prior studies with red beets report average pigment levels ranging from 40 to 200 mg BCY/100 g FT, with patented high pigment lines producing over 310 mg BCY/100 g FT (Gabelman et al., 2002; Neelwarne, 2012). Betaxanthins are also present in beets, with one study of popular cultivars reporting betaxanthin:betacyanin ratios ranging from 0.36 to 0.66 (Sapers and Hornstein, 1979). These beetroot pigment concentrations are higher than our observed values for vegetable amaranth on a per-gram basis, but amaranth’s impressive biomass yields and breeding potential offer opportunities to develop it as a natural colorant source competitive to beets. Under field conditions, amaranth can be harvested multiple times per growing season from the same original plants with individual harvest yields of up to 25.3 tonnes/ha (Shukla et al., 2006). Comparative work should, however, note the differences in molar absorptivity between amaranthin and betanin, the major betacyanins present in amaranth and beet extracts, respectively. The observed variation in pigments among the accessions we tested holds promise for vegetable amaranth breeding programs focused on increasing total betacyanin content. Further development of pigmented accessions should reference the breeding successes and genetic resources of red beets to maximize efficiency and gains in pigment yields (Gabelman et al., 2002; Neelwarne, 2012).

### Factors affecting betalain composition

Differences in betalain profiles were seen between species, with *A. cruentus* containing significantly higher percentages of celosianin II (*x̄* = 0.9%) and *A. tricolor* containing significantly higher proportions of isoamaranthin (*x̄* = 12.1%) and betanin (*x̄* = 3.1%). These trends between species are seen in other published work on *Amaranthus* betacyanins (Cai et al., 1998a, 2001a). A recent study by Spórna-Kucab et al. (2019) identified 6′-O-formyl-amaranthin and 6’-O-malonyl-amaranthin, along with isomers, in small quantities in a betacyanin-rich extract of *A. cruentus* floral tissue by using high-speed counter-current chromatography in tandem with LC–MS–MS. These minor species may have relatively little impact on amaranth’s use as a food colorant, but knowledge of their presence could prove insightful for future studies of betalain biosynthesis within the genus. Betalain composition in *Amaranthus* may be a useful chemotaxonomic marker, although we recommend additional work should be done to better characterize the betacyanin profiles of uninvestigated species and other factors potentially influencing betacyanin production such as genetics, stress, and hybridization (Packard et al., 2021).

Full spectral data from HPLC analyses were collected from 300 to 650 nm using a photodiode array detector and visualized with an isoabsorbance plot to further verify the absence of betaxanthins, which would be observed with a *λ*_max_ near 470 nm. None were detected, but a faint yellow compound (*λ*_max_ ≈ 415) determined to be betalamic acid was identified with LC–MS–MS in very minor concentrations in most accessions. Betalamic acid is a key precursor to both betacyanins and betaxanthins but is also a common degradation product of betacyanins. Given the spontaneous formation of betaxanthins upon conjugation of betalamic acid and an amino acid, extracts allowed enough time to undergo this reaction would not be representative of the plant’s *in vivo* betalain profile. The general absence or low abundance of betaxanthins among our test panel is intriguing and worthy of more research, especially compared to similar-colored carotenoids, although this finding might be partly explained by the tissues we tested.

Betacyanin composition is often explored from application-based perspectives due to the implications it is thought to have on color stability and performance in food and beverage products. Work done by Huang and Von Elbe (1986) found that, under accelerated degradation at 90°C, purified amaranthin had equal stability to betanin in oxygenated environments but less stability in anoxic environments. Another study observed nonsignificant differences between amaranthin and betanin at multiple temperatures in the presence of oxygen (Cai et al., 2001b). Amaranth-sourced betacyanins have been successfully used in various model food and beverage systems (Cai and Corke, 1999), but direct comparisons to current natural and synthetic colorants utilized by the food industry are needed to fully understand the potential of amaranth-sourced betacyanins.

The ability to make direct comparisons of extract color between the present study and other values reported in the literature for amaranth is limited due to the use of different measurement protocols. Cai and Corke (1999) and Cai et al. (1998a,b) reported colors for *Amaranthus* extracts that are substantially darker and less vivid colors that appear dark plum to gray when visualized after conversion to RGB coordinates using the Colorspace package in R (Zeileis et al., 2019). These differences appear to be due to instrumental error, as UV–Vis spectra included with the color values are very similar to spectra observed in the present study. In studies looking at other crops, however, amaranth colors described herein were similar to values from purple *Opuntia stricta* Haw. fruit, sharing low b^*^ values and high chroma (Fernández-López et al., 2013). Other colorants explored in the study such as red beet, cochineal, and anthocyanin-based extracts had higher b^*^ values, appearing more yellow, and lower chroma (Fernández-López et al., 2013). Reports on pitaya (*Hylocereus* spp.) extract colors also show close similarity to amaranth colors (Vaillant et al., 2005).

## Conclusion

Our investigation yields new findings on betalain pigments among a diverse panel of amaranth accessions from the USDA National Plant Germplasm collection and genotypes from commercial sources. In addition, our focus on betalains as potential sources of natural food colorants provided the context for optimizing high-throughput extraction and quantification methods in compliance with current US FDA food colorant standards. Simple water extractions of dried edible biomass yielded vivid magenta extracts appearing bluer than color values reported for red beet extracts, which highlights the potential of betacyanin-rich amaranth extracts as an alternative to beet-sourced food colorants. The major betacyanin of all accessions surveyed was amaranthin, but yields of betacyanins were quite variable and mainly dependent on genotype. The highest average total betacyanin yield was from accession PI 566897 (*A. cruentus*), with 478.8 mg BCY/100 g DT. Another finding was that total betacyanin yield is hard to predict based on phenotype alone, as several intensely dark-pigmented types had lower yields compared to other types with less intense pigmentation (Figure 1). Furthermore, we found that extract colors are not solely dependent on the betacyanin:betaxanthin ratio, as often proposed, since we detected no betaxanthins in our extracts. In such cases, minor uncharacterized betalains, flavonoids, or carotenoids may contribute to observed color profiles. As previously discussed, pigment yield was influenced partly by genotype and partly by growth characteristics resulting in varying stem:leaf tissue ratios. Phenotypic patterns are nonetheless good general indicators for the presence and distribution of betalains overall. Compared to beets, the use of vegetable amaranth as a food colorant could expand the hue ranges, offer greater regional diversity in sourcing raw materials, and impart less earthy flavors to final products (a common polarizing feature of beets). It is worthwhile to note that the chemically unrelated synthetic azo dye FD&C Red No. 2 (E123) “Amaranth” exists and is still used as a food colorant in certain regions, which necessitates clear product labeling for betacyanin-rich extracts derived from *Amaranthus* species.

## Dtata availability statement

The original contributions presented in the study are included in the article/Supplementary material, further inquiries can be directed to the corresponding author.

## Author contributions

This manuscript represents partial fulfilment of a graduate thesis project by JH under the supervision of CR, both of whom conceived the study. JH performed the experiments, analysed and interpreted the data, and drafted the manuscript with contributions and suggestions from CR and MV. All authors contributed to the article and approved the submitted version.

## Funding

This project was funded in part by a University of Illinois ACES-OIP Seed Grant and USDA-NIFA-AFRI grant 2019-67013-29355. Fellowship support for JH was provided by a USDA-NNF Grant 2015-38420-23707 administered through the MINDS-in-Ag Program at the University of Illinois.

## Conflict of interest

The authors declare that the research was conducted in the absence of any commercial or financial relationships that could be construed as a potential conflict of interest.

## Publisher’s note

All claims expressed in this article are solely those of the authors and do not necessarily represent those of their affiliated organizations, or those of the publisher, the editors and the reviewers. Any product that may be evaluated in this article, or claim that may be made by its manufacturer, is not guaranteed or endorsed by the publisher.
