# Supplementary material for: Amaranth as a natural food colorant source: Survey of germplasm and optimization of extraction methods for betalain pigments
Source: Front Plant Sci. 2022 Sep 21;13:932440. doi: 10.3389/fpls.2022.932440 (PMC9532763; doi:10.3389/fpls.2022.932440)

Supplementary Figure S1. Example response surface methodology (RSM) contour plots generated in Minitab 19 that can be interpreted to maximize total betacyanin content from a single extraction of freeze-dried plant material. Continuous independent variables time (minutes), temperature (°C) and mass (grams) are plotted against each other to create a contour surface showing the highest and lowest betacyanin yields.

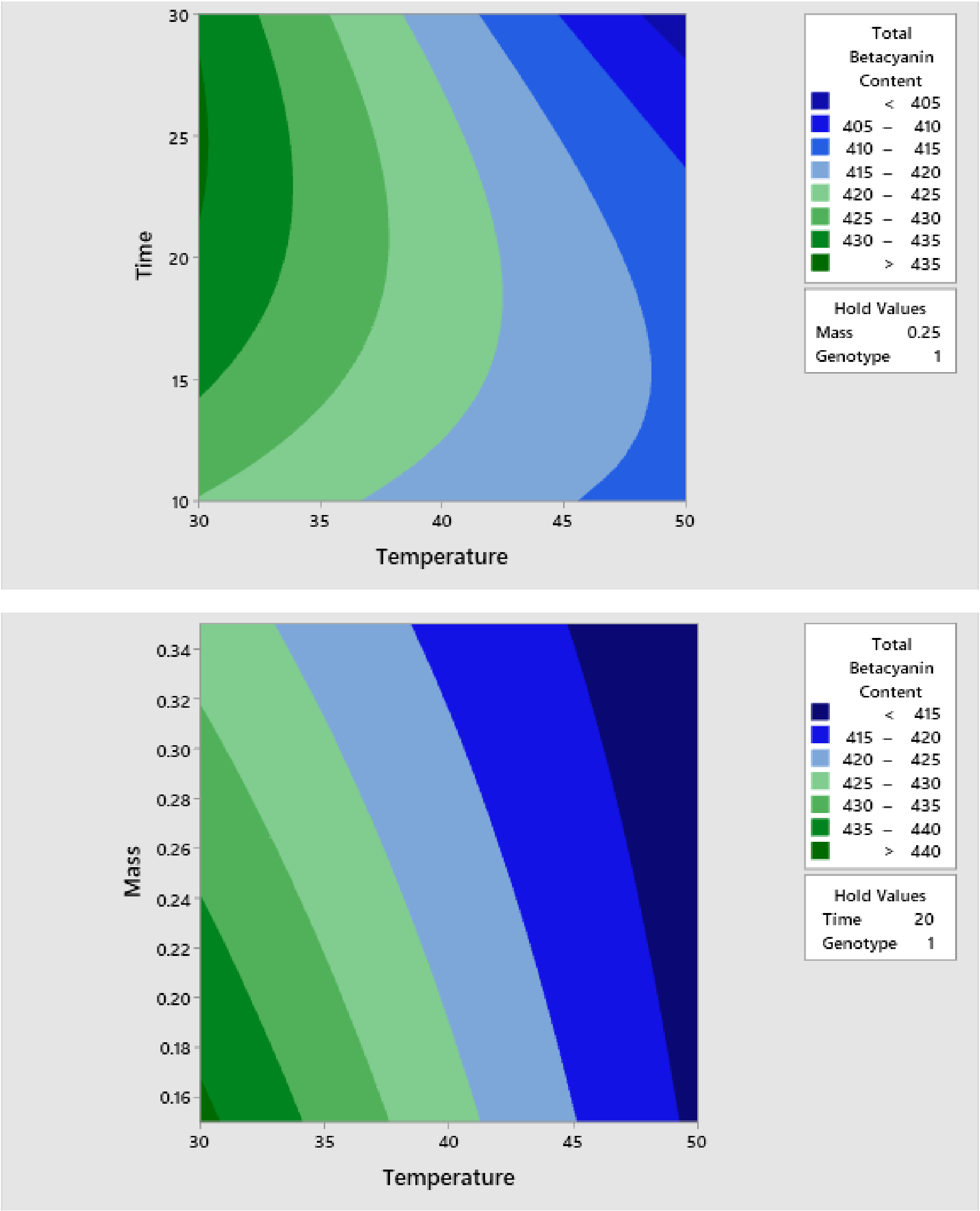

Supplementary Figure S1 continued.

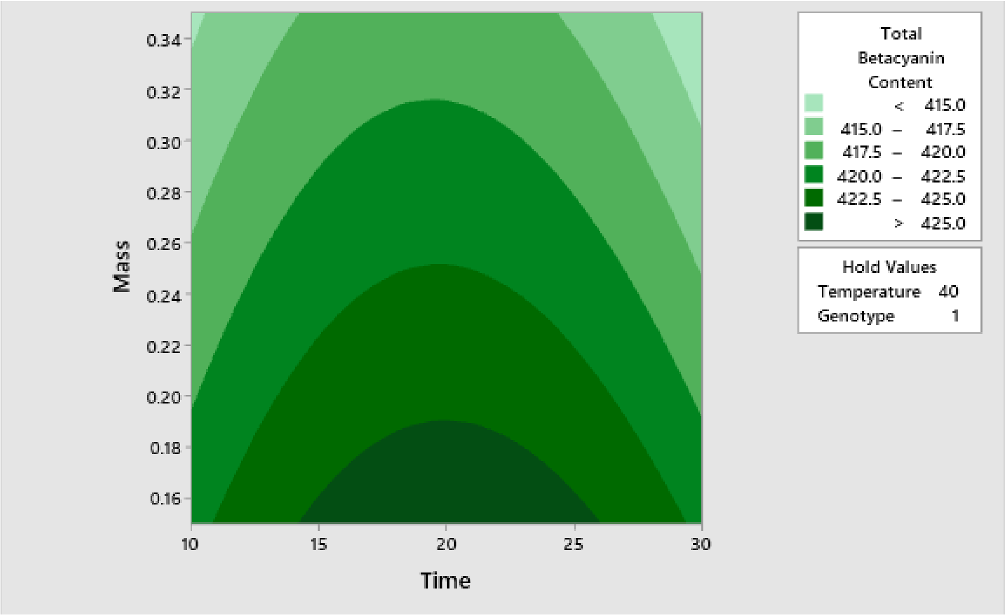

Supplement: Supplementary file 1 [file Data_Sheet_1.PDF]
