# Supplementary material for: Amaranth as a natural food colorant source: Survey of germplasm and optimization of extraction methods for betalain pigments
Source: Front Plant Sci. 2022 Sep 21;13:932440. doi: 10.3389/fpls.2022.932440 (PMC9532763; doi:10.3389/fpls.2022.932440)

Supplementary Figure S2. A) Photographs (A_{max} = 2) and B) UV-Vis spectra (A_{max} = 1) of filtered betalain-containing plant extracts and synthetic red dye #40. Extracts with exclusively betacyanins (A1 & B1, sourced for amaranth), exclusively betaxanthins (A2 & B2, sourced from yellow beets), and both betacyanins and betaxanthins (A3 & B3, sourced from red beets) are presented to show the visual and spectral differences between different combinations of betalains. Red 40 (A4 & B4) is included as a reference for visual comparison of color hue and chroma (saturation).

A

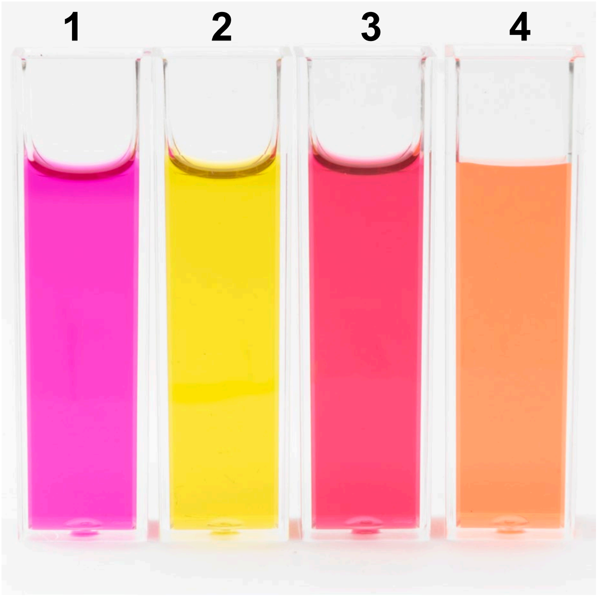

B

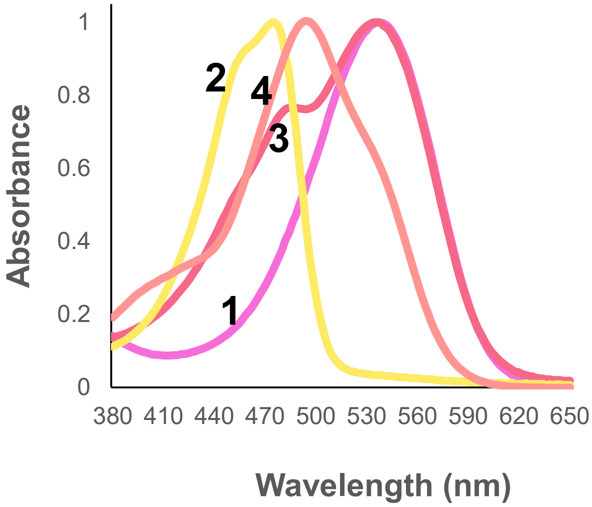

Supplement: Supplementary file 2 [file Data_Sheet_2.PDF]
