# Supplementary material for: Amaranth as a natural food colorant source: Survey of germplasm and optimization of extraction methods for betalain pigments
Source: Front Plant Sci. 2022 Sep 21;13:932440. doi: 10.3389/fpls.2022.932440 (PMC9532763; doi:10.3389/fpls.2022.932440)

Supplementary Figure S3. Micrographs of stem (A) and leaf (B) tissue sections of *A. cruentus* PI 689689 showing the accumulation of betacyanins in epidermal tissues.

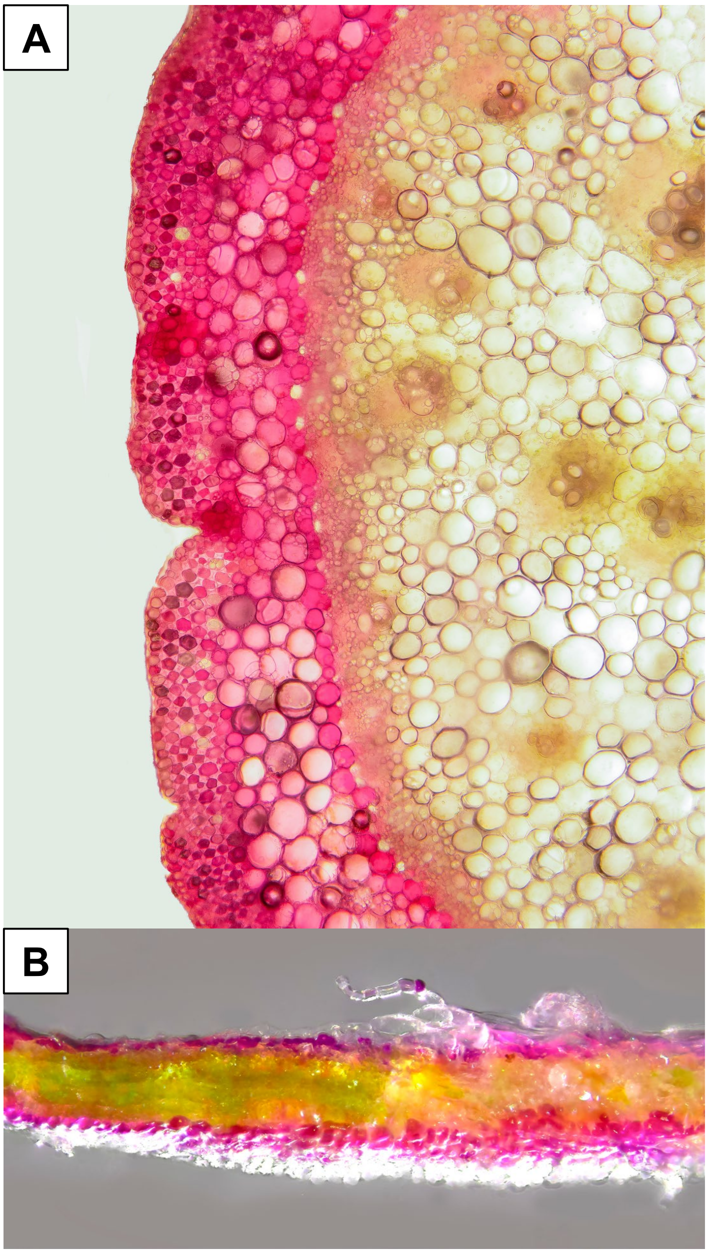

Supplement: Supplementary file 3 [file Data_Sheet_3.PDF]
