# Supplementary material for: Amaranth as a natural food colorant source: Survey of germplasm and optimization of extraction methods for betalain pigments
Source: Front Plant Sci. 2022 Sep 21;13:932440. doi: 10.3389/fpls.2022.932440 (PMC9532763; doi:10.3389/fpls.2022.932440)

Supplementary Figure S4. Photographs of mature plants of *A. tricolor* accessions Ames 25153 (A) and the cultivar' Aurora Yellow' from Baker Creek Seeds (B). Both cultivars present unique yellow pigmentation from carotenoids in the absence of chlorophylls.

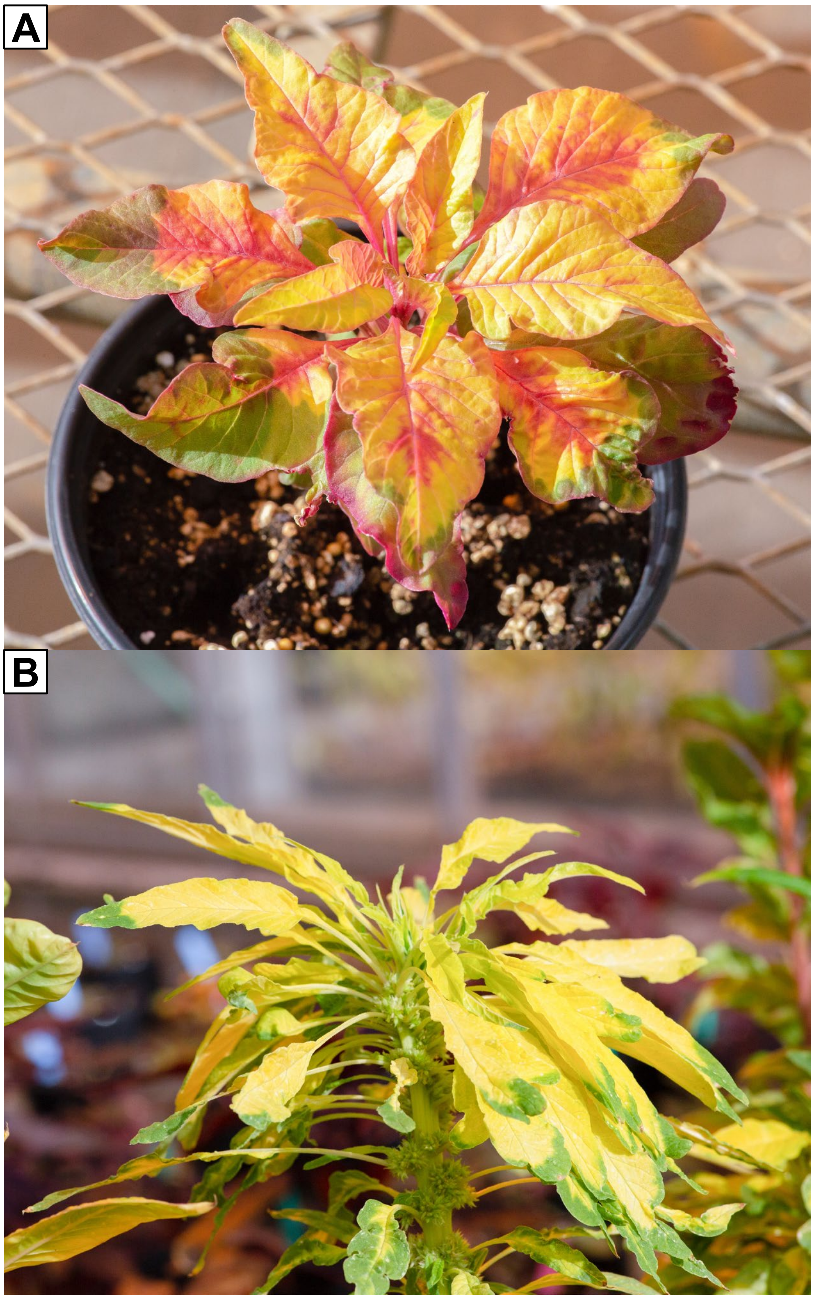

Supplement: Supplementary file 4 [file Data_Sheet_4.PDF]
